# Severe Hypertriglyceridemia Induced Pancreatitis in Pregnancy

**DOI:** 10.1155/2014/485493

**Published:** 2014-06-03

**Authors:** Natasha Gupta, Seema Ahmed, Lemuel Shaffer, Paula Cavens, Josef Blankstein

**Affiliations:** Department of Obstetrics and Gynecology, Mount Sinai Hospital, 1500 S California Avenue, Chicago, IL 60608, USA

## Abstract

Acute pancreatitis caused by severe gestational hypertriglyceridemia is a rare complication of pregnancy. Acute pancreatitis has been well associated with gallstone disease, alcoholism, or drug abuse but rarely seen in association with severe hypertriglyceridemia. Hypertriglyceridemia may occur in pregnancy due to normal physiological changes leading to abnormalities in lipid metabolism. We report a case of severe gestational hypertriglyceridemia that caused acute pancreatitis at full term and was successfully treated with postpartum therapeutic plasma exchange. Patient also developed several other complications related to her substantial hypertriglyceridemia including preeclampsia, chylous ascites, retinal detachment, pleural effusion, and chronic pericarditis. This patient had no previous family or personal history of lipid abnormality and had four successful prior pregnancies without developing gestational hypertriglyceridemia. Such a severe hypertriglyceridemia is usually seen in patients with familial chylomicronemia syndromes where hypertriglyceridemia is exacerbated by the pregnancy, leading to fatal complications such as acute pancreatitis.

## 1. Introduction

Hypertriglyceridemia associated with pregnancy occurs due to normal, physiological changes of pregnancy, involving increased sex hormone levels. Gestational hypertriglyceridemia is usually not very severe and does not result in medical or obstetric complications. This physiological increase in lipid levels can be exacerbated by an underlying abnormality of lipid metabolism, leading to catastrophic consequences such as acute pancreatitis. We report such a case of severe gestational hypertriglyceridemia that leads to acute pancreatitis and other associated complications, even in absence of any underlying genetic defects of lipid metabolism.

## 2. Case Presentation

We report a 32-year-old Hispanic female gravida five para four, at 38-week and four-day gestational age, who presented with severe epigastric pain, of one-day duration. She complained of several episodes of emesis but denied any uterine contractions, fever, chills, or diarrhea. She had no history of similar pain in the past. She had no history of alcohol use, drug intake, gallstones, pancreatitis, or similar episode in previous pregnancies. Upon examination, her blood pressures were elevated to systolic 150–160s and diastolic 90s. Other vital signs were normal. Her cervical os was dilated to 2 cms dilatation, with 50% effacement and −3 station. Abdomen was noted to be nontender, with no evidence of appendicitis or cholecystitis. Her laboratory studies showed sodium 121 meq/L (normal range: 136–146 meq/L), potassium 3.1 meq/L (normal range: 3.5–5.1 meq/L), lactate 2.3 mmol/L (normal range: 0.5–2.2 mmol/L), amylase 1617 U/L (normal range: 16–96 U/L), lipase 1330 U/L (normal range: 22–51 U/L), and normal liver function tests (Table 1). Urinalysis showed 2+ proteinuria (100 mg/dL) and ketones 150 mg/dL. Thus, a diagnosis of mild preeclampsia and acute pancreatitis was made based on patient's symptomatology and elevated amylase and lipase levels. She was admitted for conservative management of acute pancreatitis and induction of labor for preeclampsia. She received magnesium sulfate for seizure prophylaxis and intravenous hydration as resuscitative measure. During the course of this treatment, she spontaneously progressed to a cervical dilatation of 5 cms, but her condition deteriorated as suggested by maternal and fetal tachycardia as well as metabolic acidosis on arterial blood gas analysis. Repeat laboratory studies were significant for lactate of 7 mmol/L and calcium 4.5 mg/dL (normal range: 8.4–10.2 mg/dL). Her APACHE II score was placed at 13 and Ranson's at 3, thus predicting maternal mortality from acute pancreatitis at 18%.

**Figure 1:**
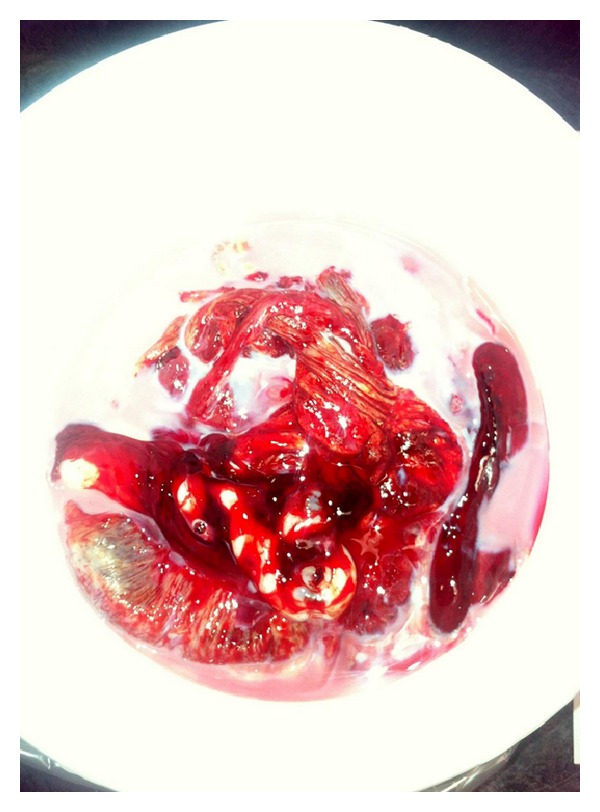
Placenta covered with milky fluid suggestive of severely increased serum lipid levels.

**Figure 2:**
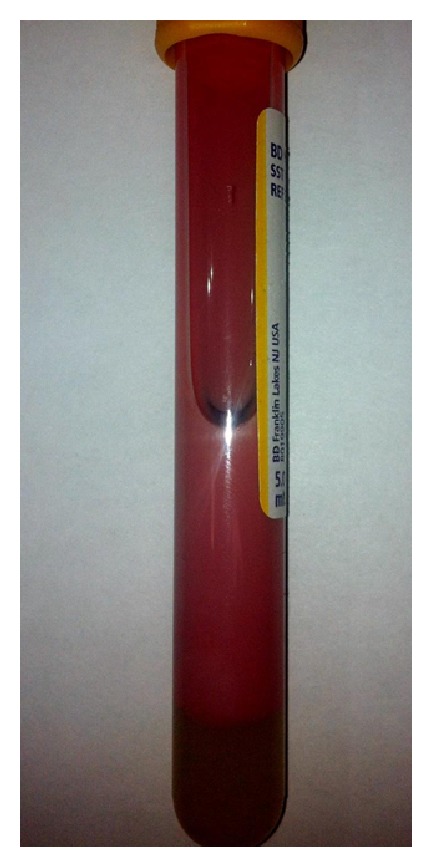
Patient's serum sample with lipemic appearance on day 1 of admission.

An immediate cesarean delivery was recommended by maternal-fetal medicine and intensive care unit. During surgery, intraperitoneal fluid was noted to be milky-pink and lipemic. Similarly, placenta appeared to be covered with milky fluid (Figure 1). A serum sample drawn from the patient also had similar appearance (Figure 2). Her lipid profile was examined which showed the triglyceride concentration of 12,570 mg/dL and total cholesterol of 1,067 mg/dL. At this time, a diagnosis of hypertriglyceridemia induced acute pancreatitis was considered. Patient also had severe edematous pancreatitis, moderate ascites, mild splenomegaly, and mild diffuse fatty infiltration of liver on computed tomography, although there was no evidence of necrosis, thrombosis, or pseudocyst of the pancreas.

**Figure 3:**
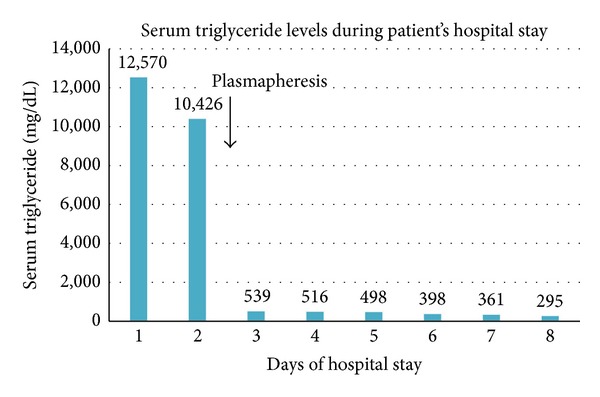
Decline in serum triglyceride level after plasmapheresis was performed on day 2 of admission.

Plasmapheresis was used as the modality of treatment for pancreatitis secondary to severe hypertriglyceridemia. Patient underwent one session of plasmapheresis and was also started on fibrate derivatives as well as low fat, restricted calorie clear liquid diet. Her triglyceride levels decreased to 498 mg/dL by day 5 of admission (Figure 3) and total cholesterol level decreased to 179 mg/dL, with this treatment. She was continued on a strict low fat, low calorie solid diet. Patient also developed pleural effusion and chronic pericarditis during the course of her treatment. She complained of blurring of vision on day 15 of admission and was diagnosed with retinal detachment, for which she received outpatient surgical treatment. She was discharged after 15 days, on strict low fat, low calorie diet and fenofibrates. Her triglycerides returned to normal levels on follow-up visit.

## 3. Discussion

Acute pancreatitis secondary to severe hypertriglyceridemia is a rare, but lethal condition. More commonly, acute pancreatitis is known to be caused by biliary tract disease such as gall stone pancreatitis or non-biliary tract disease such as alcoholism, medication use, and hypertriglyceridemia. It is a rare complication in pregnancy, but when it occurs due to any cause, it leads to high maternal and fetal morbidity and mortality.

Hypertriglyceridemia is defined as triglyceride levels more than 150 mg/dL. It can be classified into mild (150–199 mg/dL), moderate (200–999 mg/dL), severe (1,000–1,999 mg/dL), or very severe (>2,000 mg/dL) disease. Etiology of hypertriglyceridemia can be classified into primary and secondary causes. Primary hypertriglyceridemia can be due to chylomicronemia syndrome [1, 2], associated with underlying deficiency of lipoprotein lipase (LPL) or apoprotein C-II [1, 3], whereas secondary hypertriglyceridemia is associated with diabetes mellitus, chronic drug abuse, exogenous estrogen or tamoxifen use, and pregnancy [2]. Pregnancy is known to be associated with mild increase in triglyceride and cholesterol levels [4] caused by elevated estrogen levels, which lead to downregulation of LPL gene expression, thus decreasing the LPL activity and decreased clearance of VLDL-C. Increased estrogen levels of pregnancy also lead to increased synthesis of triglycerides and very low density lipoproteins (VLDL) by liver [1]. Despite these alterations in the lipid profile, triglyceride levels rarely exceed 300 mg/dL by third trimester. Severe gestational hypertriglyceridemia can occur if patient has an underlying defect in lipid metabolism, which exacerbates the physiological hypertriglyceridemia of pregnancy [5].

We report such a patient here that developed very severe gestational hypertriglyceridemia, without any history of abnormality in lipid profile during pregnancy or otherwise. She developed acute pancreatitis and other complications as a result of severely elevated triglycerides. Triglycerides are broken into toxic, free fatty acids by pancreatic lipases, which cause lipotoxicity and thus acute pancreatitis. Severity of acute pancreatitis depends on the triglyceride level, pancreatic lipase activity, efficiency of clearing free fatty acids from serum, and severity of underlying pancreatic injury. Risk of developing acute pancreatitis increases progressively when triglyceride levels exceed 500 mg/dL. It also increases as pregnancy advances, with 19% risk in first trimester, 26% in second trimester, 53% in third trimester, and 2% in postpartum period [6].

Acute pancreatitis is suspected in pregnancy when patient has nonobstetrical abdominal pain, especially epigastric pain, associated with nausea, vomiting, and fevers. Also, underlying cause of pancreatitis should be identified with detailed history about alcohol use, drug abuse, medications, and features of symptomatic cholelithiasis. Personal or family history of hyperlipidemia should prompt measurement of lipid levels, as a possible etiology of acute pancreatitis. Signs of severe hypertriglyceridemia include lipemic appearance of blood sample, xanthomas over external surfaces of arms, legs, and buttocks, lipemia retinalis, and hepatosplenomegaly. Other known complications of acute pancreatitis include pancreatic pseudocyst formation, pancreatic necrosis, shock, hypocalcemia, and preeclampsia or eclampsia. Our patient presented with severe epigastric pain and several episodes of emesis. Differential diagnosis of labor, appendicitis, cholecystitis, and other surgical causes of abdominal pain were considered but were ruled out based on her symptomatology, examination findings, and laboratory studies. In our patient, acute pancreatitis was diagnosed early based on her symptoms and serum amylase and lipase levels, but an underlying etiology could not be identified, until chylous ascites was noted intraoperatively. Our patient developed chylous ascites, pleural effusion, chronic pericarditis, and retinal detachment as sequel to acute pancreatitis and severe hypertriglyceridemia.

This case illustrates the importance of measuring lipid profile early in pregnancy, especially in presence of family or personal history of preexisting lipid abnormality or secondary risk factors like diabetes mellitus, drug use, and so forth. If patient is diagnosed with, or is at risk for, hypertriglyceridemia, treatment should be aimed at prevention of acute pancreatitis in pregnancy. This can be achieved by initiating strict low fat, low calorie diet and nutritional support with medium chain triglycerides as well as omega-3 fatty acids [7, 8]. Fasting lipid profiles are monitored intensely and one or more lipid lowering medications like fibrates, statins, niacin, and gemfibrozil [9] can be added when high levels are noted. A well planned, preterm cesarean delivery can be arranged for patients with very high levels of lipids, previous history of hypertriglyceridemic acute pancreatitis, or personal history of chylomicronemia syndrome [8]. If acute pancreatitis develops despite these preventative and therapeutic measures, an early diagnosis and intensive treatment can prevent maternal and fetal morbidity and mortality. Maternal mortality is usually predicted at 20% and fetal mortality at 50% with acute pancreatitis secondary to severe hypertriglyceridemia [1]. We managed our patient conservatively with intravenous hydration, adequate analgesia, and nil per oral once acute pancreatitis was diagnosed, while simultaneously initiating induction of labor for management of preeclampsia. Hypertriglyceridemia was managed with a combination of plasmapheresis and low fat and low calorie diet, in our patient. Different authors have proposed several different modalities for the treatment of hypertriglyceridemia, which includes plasmapheresis [10, 11], infusion of heparin [12, 13], and intravenous infusion of insulin and glucose [1, 10]. Some authors have reported successful treatment with the use of combination of different modalities like plasmapheresis with heparin infusion [14] or combined infusion of heparin and insulin. Heparin and insulin infusion are low cost alternatives to plasmapheresis but the first modality of choice for treatment of hypertriglyceridemia still remains conservative approach with nutritional and pharmacological methods [11]. Nutritional methods include dietary restriction of fatty meal and introduction of medium chain triglycerides and omega-3 fatty acids in diet, while pharmacological methods include lipid lowering medications like statins, fibrates, and so forth [11]. Plasmapheresis or lipid apheresis is especially indicated when (a) patient is refractory to nutritional and pharmacological approaches, (b) serum triglycerides exceed 1000 mg/dL, (c) serum lipase is three times the upper limit of normal, (d) patient develops hypocalcemia, (e) lactic acidosis occurs, and (f) there is worsening inflammation and organ dysfunction. Our patient met four out of these six criteria, which prompted initiation of plasmapheresis. Plasmapheresis can be stopped once serum triglycerides drop below 500 mg/dL. Albumin or fresh frozen plasma may be used for replacement fluid, and heparin is used for anticoagulation during therapeutic plasma exchange. When plasmapheresis is unavailable or contraindicated, intravenous infusion of regular insulin and 5% dextrose can be used. Blood sugar is maintained between 150 and 200 mg/dL during this therapy. Insulin and heparin infusions act by stimulating LPL activity, which then removes triglycerides from the plasma [1, 15].

After the phase of acute pancreatitis had subsided and lipid levels were brought under adequate control, we managed our patient as outpatient, with close follow-up of lipid levels, dietary restrictions, and fenofibrates. Most authors recommend low fat and low calorie diet to be continued for a long term [2], with dietary supplementation of medium chain triglycerides [2] and omega-3 fatty acids. Some authors also started treatment with one or more lipid lowering medications like niacin, statins, fibrates, or gemfibrozil [1]. Our patient presented some unusual features of interest as she had no prior personal or family history of hypertriglyceridemia and never reported gestational hyperlipidemia or its associated complications in the previous four pregnancies which were all well documented, with adequate prenatal care, at our institution. We were unable to identify familial cause of her hypertriglyceridemia like chylomicronemia syndrome and could only attribute it to gestational hyperlipidemia. Also, her serum triglycerides and cholesterol levels exceeded well above those recorded in the literature. We successfully managed our patient with early diagnosis of acute pancreatitis, prompt termination of pregnancy on worsening of her condition, and timely application of plasmapheresis once severe hypertriglyceridemia was recognized.

## 4. Conclusion

The diagnosis of hypertriglyceridemia induced acute pancreatitis can be delayed due to its low incidence and thus low suspicion by modern obstetrician. Any nonobstetrical abdominal pain should prompt evaluation for acute pancreatitis and its causative factors like hyperlipidemia, especially since gestational hyperlipidemia is becoming a more common entity than previously believed. Timely diagnosis and management of both acute pancreatitis and hypertriglyceridemia and their secondary causes can help prevent maternal and fetal morbidity and mortality.

## Figures and Tables

**Table 1:** Patient's laboratory data on admission.

	Result	Normal ranges		Result	Normal ranges
Triglyceride	12,570	50–150 mg/dL	Sodium	121	136–146 meq/L
Cholesterol	1,067	<200 mg/dL	Potassium	3.1	3.5–5.1 meq/L
Amylase	1,617	16–96 U/L	Calcium	8	8.7–10.3 mg/dL
Lipase	1,330	22–51 U/L	Lactate	2.3	0.5–2.2 mmol/L
AST	14	10–42 IU/L	Hemoglobin	11.6	12.0–16.0 g/dL
ALT	13	10–60 IU/L	Leucocyte	10.9	4.5–11 cells/*μ*L
Creatinine	0.10	0.5–1.10 mg/dL	TSH	0.32	0.34–5.6 IU/mL

AST: aspartate aminotransferase; ALT: alanine aminotransferase; TSH: thyroid stimulating hormone.
